# Pseudo‐Observation Approach for Length‐Biased Cox Proportional Hazards Model

**DOI:** 10.1002/bimj.70094

**Published:** 2025-10-30

**Authors:** Mahboubeh Akbari, Najmeh Nakhaei Rad, Ding‐Geng Chen

**Affiliations:** ^1^ Department of Statistics University of Pretoria Pretoria South Africa; ^2^ College of Health Solution Arizona State University Phoenix Arizona USA

**Keywords:** Cox proportional hazards model, generalized estimation equation, length‐biased data, ‐observation

## Abstract

Pseudo‐observations are used to estimate the expectation of a function of interest in a population when survival data are incomplete due to censoring or truncation. Length‐biased sampling is a special case of a left‐truncation model, in which the truncation variable follows a uniform distribution. This phenomenon is commonly encountered in various fields such as survival analysis and epidemiology, where the event of interest is related to the length or duration of an underlying process. In such settings, the probability of observing a data point is higher for longer lengths, leading to biased sampling. The goal of this paper is to apply pseudo‐observations to estimate the regression coefficients in the Cox proportional hazards model under length‐biased right‐censored (LBRC) data. We assess the accuracy and efficiency of two approaches that differ in their generation of pseudo‐observations, comparing them with two prominent standard methods in the presence of LBRC data. The results demonstrate that the two proposed pseudo‐observation methods are comparable to the standard methods in terms of standard error, with advantages in providing confidence intervals that are closer to the nominal level in large sample sizes and specific scenarios. Additionally, although length‐biased data are a special case of left‐truncated data, they must be addressed separately by utilizing the information that the left‐truncation variable follows a uniform distribution, as the simulation results show. We also establish the consistency and asymptotic normality of one of the proposed estimators. Finally, we applied the method to analyze a real dataset from LBRC.

## 1 Introduction

In survival analysis, the focus is on examining the time between an initiating event and a specific or terminating event, such as the time from the onset of a disease to relapse or death due to the disease. Examples include the time to Human Immunodeficiency Virus (HIV) infection, the time from the onset of Alzheimer's or Parkinson's disease to the development of symptoms, or the time from an organ transplant to organ rejection or patient death. The variable we measure, $\tilde{T}$, is called the survival time, event time, or failure time. On some occasions, however, observations may not be representative of the original data, and the selection of an observation is proportional to its length or duration. This is known as length‐biased sampling. In the context of prevalent cohort designs, length bias occurs when individuals with longer disease durations are more likely to be included in the study population than those with shorter durations. Length‐biased data can be viewed as a special case of left‐truncated data, where the truncation variable follows a uniform distribution, known as the stationarity assumption. In this sampling design, an individual qualifies for inclusion in the sampling population if they survive until the recruitment time. This means that the survival time of the ith subject, ${\tilde{T}}_{i}$, is greater than the time from disease onset to recruitment time, ${\tilde{A}}_{i}$, that is, ${\tilde{T}}_{i}|{\tilde{T}}_{i}>{\tilde{A}}_{i}$. In addition to sampling bias, some individuals may be lost to follow‐up or drop out of the study before the terminating event occurs. Therefore, in this case, we are dealing with length‐biased right‐censored (LBRC) data.[Supplementary-material bimj70094-supl-0001]

As an example of an LBRC dataset, the Canadian Study of Health and Aging (CSHA) is one of the largest epidemiological studies of dementia. The participants from this cohort were screened for dementia in 1991. As indicated by Wolfson et al. (2001), the CSHA includes left‐truncated observations because survival data were collected from a prevalent cohort of patients with dementia who had not experienced the failure event (death) at the recruitment time. It has been shown that the disease incidence in the CSHA data is stable over time by the formal test of Addona and Wolfson (2006). Another example, which is publicly available, is the Channing House dataset (Hyde 1980). This data were gathered at a retirement center in Palo Alto, California, from 1964 to 1975. The observed survival times are left‐truncated because only individuals who had lived long enough to enter the retirement center were included in the data. To reflect the nature of length‐biased sampling, only elderly individuals aged 65 years or older were considered the target group of interest. In these applications, a primary interest is to assess the effect of different covariates on the hazard or risk of the event occurring.

LBRC data can pose challenges in statistical analysis. First, for estimating the survival function, standard methods such as Kaplan–Meier estimator (Kaplan and Meier 1958) are inappropriate and prone to overestimation (Wolfson et al. 2001). Second, the Cox proportional hazards (Cox PH) model is not invariant under LBRC data. This means that the structure of the regression model for the target population may differ from the structure of the regression model for the collected sample when the data are subjected to LBRC. This introduces challenges in correctly modeling and interpreting the relationship between the hazard rate and risk factors in the Cox PH model when analyzing length‐biased data. To address these issues, several methods have been proposed in the literature (see reviews by Shen et al. 2017; Akbari et al. 2024). These approaches aim to develop appropriate statistical techniques to provide reliable estimates of the survival function and to properly model the relationship between survival time and covariates in the presence of length‐biased sampling and right‐censoring.

An alternative approach to handling incomplete observations, such as right‐censored, interval censored, left‐truncated, and LBRC data, in survival analysis is the pseudo‐observation approach, which has become increasingly popular in recent years. Andersen et al. (2003) proposed a general approach to censored data regression based on pseudo‐observations. The pseudo‐observations, derived from jackknife theory, enable the use of standard regression methods such as the generalized estimating equations (GEEs) approach (Liang and Zeger 1986) to estimate regression coefficients. Hence, GEE provides unbiased estimates of the regression parameters, and the standard deviation of the coefficients can be estimated using a sandwich estimator (Klein 2006). This approach has been applied to various survival analysis models, including regression models for the cumulative incidence functions in competing risks (Andersen and Klein 2007; Graw et al. 2009; Jacobsen and Martinussen 2016; Klein 2006; Klein and Andersen 2005), state occupation probabilities in multistate models (Andersen et al. 2003; Andersen and Klein 2007), the restricted mean survival time (Andersen et al. 2004), and the survival function (Klein et al. 2007). A summary of different methods for survival analysis based on the pseudo‐observation approach is presented by Andersen and Perme (2010).

Grand et al. (2019) proposed two different types of pseudo‐observations under left‐truncated data and explored their performance in a simulation study to estimate log hazard ratios. In the literature, methods that treat length bias as random left truncation, with the truncating distribution assumed to be unknown, generally cause very little loss of information (Asgharian et al. 2002; Huang and Qin 2011). Although Grand et al. (2019) examined pseudo‐observations under left truncation, their application to length‐biased data remained unexplored and may introduce bias. Therefore, using pseudo‐observations derived from a survival estimator function specifically designed for LBRC data may potentially capture more information present in the data compared to those proposed by Grand et al. (2019). Moreover, we compare the pseudo‐observation methods with two prominent standard approaches proposed by Qin and Shen (2010) and Huang and Qin (2012) for estimating the coefficients of Cox PH model under LBRC data. To our knowledge, there are no results in the literature establishing the asymptotic properties of pseudo‐observation‐based estimators for the Cox PH model under LBRC settings. This article addresses that gap by extending the concept of pseudo‐observations to length‐biased data and investigating its asymptotic properties.

The outline of this paper is as follows. In Section 2, we provide some notations and preliminaries in the length‐biased context and review the nonparametric estimator of the survival function. In this section, we also introduce the pseudo‐observations. Section 3 develops two estimators for the Cox PH model coefficients under LBRC data using the Jackknife pseudo‐observation approach. Section 4 presents the asymptotic properties of one of the proposed estimator, including consistency and asymptotic normality. In Section 5, the performance of the proposed estimators is compared with existing methods through simulations studies. The application of the proposed methods to a real‐world LBRC dataset is presented in Section 6. Section 7 provides a brief summary of the main findings, discusses their implications, and offers concluding remarks. All proofs are postponed to the Appendix.

## 2 Notations and Preliminaries

### 2.1 Length‐Biased Data

Let V represent the time from recruitment to death or censoring, and let C denote the censoring time from enrollment to the occurrence of censoring. Here, the censoring is informative because the censoring variable A+C and survival time T=A+V share the same A, where A is the time from onset to recruitment. We assume that F(·) and S(·)=1−F(·) denote the cumulative distribution and survival function of the unbiased survival time $\tilde{T}$, respectively, and G denotes the survival function of C. We further assume that $\tilde{T}$ and $\tilde{A}$ are conditionally independent given covariates $\tilde{X}$, where $\tilde{X}={\left({\tilde{X}}_{1},\text{…},{\tilde{X}}_{p}\right)}^{⊤}$ is a p×1 vector of baseline covariates. The joint distribution of (A,T), denoted as $f_{\left(A,T\right)}$, is given by (Lancaster 1992)

(1) $$\begin{array}{c} f_{\left(A,T\right)}\left(a,t\right)=\frac{f(t)}{\mu },\;t>a>0, \end{array}$$

where f(·) is the density function of the unbiased survival time $\tilde{T}$, and $\mu =E\left(\tilde{T}\right)=\int_{0}^{\infty }tf\left(t\right)dt$. The collected sample can be represented as $O_{i}=\left(A_{i},Y_{i},\Delta_{i},X_{i}\right),\;i=1,\text{…},n$, where $Y_{i}=A_{i}+\min\left(V_{i},C_{i}\right)=A_{i}+{\tilde{V}}_{i}$, $\Delta_{i}=I\left(V_{i}\leq C_{i}\right)$, and $X_{i}$ is the corresponding covariate vector in the biased population.

The problem of finding a nonparametric estimator of the survival function in the presence of LBRC data has been addressed by several authors, including Huang and Qin (2011), Luo and Tsai (2009), Vardi (1982a), and Wang et al. (2017). Vardi (1982a) derived the nonparametric maximum likelihood estimator (NPMLE) of the survival function under LBRC data, which does not have a closed form and needs to be obtained via the expectation‐maximization (EM) algorithm (Vardi 1989). Let $F_{LB}$ denote the cumulative distribution function of the length‐biased lifetime T, which can be obtained by maximizing

$$\begin{array}{c} L\left(F_{LB}\right)=\prod_{i=1}^{n}F_{LB}\left(dy_{i}\right)\prod_{i=1}^{n}\int_{y\geq A_{i}}\frac{1}{y}F_{LB}\left(dy\right). \end{array}$$

Let ${\hat{F}}_{LB}$ be the maximizer of this likelihood, and define ${\hat{S}}_{LB}=1-{\hat{F}}_{LB}$. The NPMLE for S, denoted by ${\hat{S}}^{V}$, is then

(2) $$\begin{array}{c} {\hat{S}}^{V}\left(y\right)=\frac{\int_{y}^{\infty }\frac{1}{x}{\hat{S}}_{LB}\left(dx\right)}{\int_{0}^{\infty }\frac{1}{x}{\hat{S}}_{LB}\left(dx\right)},\;y>0. \end{array}$$

Vardi and Zhang (1992) established the asymptotic properties of ${\hat{S}}^{V}$. Later, some attempts were made to propose simpler estimators for the survival function in this setting that are as efficient as Vardi's estimator. Wang et al. (2017) proposed a product‐limit estimator that does not lose much efficiency compared to the Vardi's NPMLE and has a closed‐form expression. Their estimator is in accordance with He and Zhou (2020), who derived the same result from the composite conditional likelihood method. Consider the risk set $R_{i}\left(t\right)=I\left(A_{i}\leq t\leq Y_{i}\right)+\Delta_{i}I\left({\tilde{V}}_{i}\leq t\leq Y_{i}\right)$ and functions

(3) $$\begin{array}{c} H_{n}\left(t\right)=\frac{1}{n}\sum_{i=1}^{n}\Delta_{i}I\left(Y_{i}\leq t\right), \end{array}$$

and

(4) $$R_{n}\left(t\right)=\frac{1}{2n}\sum_{i=1}^{n}R_{i}\left(t\right),$$

where $nH_{n}\left(t\right)$ counts the number of deaths up to time t, and $nR_{n}\left(t\right)$ estimates the number of individuals at risk at time t given the LBRC observation. Based on these definitions, the product‐limit estimator of Wang et al. (2017) is derived as follows:

(5) $$\begin{array}{c} {\hat{S}}^{W}\left(t\right)=\prod_{u\leq t}\left(1-\frac{dH_{n}\left(u\right)}{R_{n}\left(u\right)}\right). \end{array}$$

The asymptotic properties of ${\hat{S}}^{W}\left(\cdot \right)$, including its consistency and asymptotic normality, have been studied in Wang et al. (2017). Moreover, the convergence rate of the strong representation of the corresponding process associated with ${\hat{S}}^{W}\left(\cdot \right)$ has been further investigated by Akbari et al. (2024). Define

$$\begin{array}{c} H\left(t\right)=E\left[\Delta I\left(Y\leq t\right)\right],\;\text{and}\;R\left(t\right)=\frac{1}{2}\;E\left[R_{i}\left(t\right)\right], \end{array}$$

where H(·) and R(·) can be consistently estimated by their empirical counterparts $H_{n}\left(\cdot \right)$ and $R_{n}\left(\cdot \right)$, as defined in (3) and (4), respectively. Note that H(·) is a subdistribution function corresponding to F(·), representing the proportion of uncensored failure events before time t under length‐bias sampling. We can express H(t) and R(t) in terms of the true survival function S(t) as follows:

(6) $$\begin{array}{c} dH\left(t\right)=\mu^{-1}w\left(t\right)dF\left(t\right),\;\text{and}\;R\left(t\right)=\mu^{-1}S\left(t\right)w\left(t\right), \end{array}$$

where μ is the mean of the survival time $\tilde{T}$ and $w\left(t\right)=\int_{0}^{t}G\left(u\right)du$.

In the following proposition, we present a representation for ${\hat{S}}_{W}\left(\cdot \right)$ that facilitates the study of asymptotic behavior of the regression coefficients estimated using the pseudo‐observation approach in the GEE framework.

Proposition 2.1 Let $u_{F}$ be the upper bound of the support of the distribution function F(t), and define the function $\phi_{i}\left(t\right)$ as

(7) $$\begin{array}{cc} \phi_{i}\left(t\right)=\frac{\Delta_{i}I\left(Y_{i}\leq t\right)}{R(Y_{i})}- & \frac{1}{2}\int_{0}^{t}R^{-2}\left(u\right)R_{i}\left(u\right)dH\left(u\right). \end{array}$$

Then, for $0\leq t\leq b<u_{F}$, we have

$$\begin{array}{c} {\hat{S}}^{W}\left(t\right)=S\left(t\right)-n^{-1}\sum_{i=1}^{n}S\left(t\right)\phi_{i}\left(t\right)+L_{n}\left(t\right), \end{array}$$

where $L_{n}\left(t\right)=O_{p}\left(n^{\frac{-3}{2}}{\left(\log n\right)}^{2}\right)$.

See the Appendix.□

As observed, the order of the reminder term in Proposition 2.1, that is, $L_{n}\left(t\right)=O_{p}\left(n^{\frac{-3}{2}}{\left(\log n\right)}^{2}\right)$ is sufficiently small to enable the derivation of the asymptotic properties of the proposed estimator in the next section. This is particularly important because the term $n{\hat{S}}^{W}\left(t\right)$ appears in the expression for the pseudo‐observations. Moreover, it can be verified that $E(\phi_{i}\left(t\right))=0$.

### 2.2 Pseudo‐Observations for LBRC data

In this subsection, we first introduce the jackknife pseudo‐observation approach and then extend it to the LBRC setting. Let $\theta =E(\psi \left(\tilde{T}\right))$ be the parameter of interest for some measurable function ψ(·), and let $\hat{\theta }$ denote an estimator of θ. The jackknife is a nonparametric technique used to estimate the bias and variance of an estimator. It was independently developed by Quenouille (1949) and Tukey (1958), who observed that a bias‐corrected jackknife estimate could be expressed as the mean of n pseudo‐observations, defined for the ith sample observation as

(8) $$\begin{array}{c} {\tilde{\theta }}_{i}=n\hat{\theta }-\left(n-1\right){\hat{\theta }}^{\left(-i\right)},\;i=1,\text{…},n, \end{array}$$

where $\hat{\theta }$ is the estimator for θ computed from the full dataset, and ${\hat{\theta }}^{\left(-i\right)}$ is the “leave‐one‐out” estimator for θ based on all observations except the ith. The jackknife pseudo‐observations ${\tilde{\theta }}_{i}$ serve as pseudo‐observations of $\psi (\tilde{T})$ and can be treated as independent and identically distributed random variables. There are also several variations of the jackknife, such as the balanced jackknife (Shao and Wu 1989) and the delete‐d jackknife (Wu 1986), which can provide refinements under specific conditions. A comprehensive introduction to different aspects of jackknife pseudo‐observations in survival analysis is provided by Andersen et al. (2003), who proposed a method based on the pseudo‐observations. If the parameter of interest is the survival function at a fixed time point t, that is, $S\left(t\right)=E(I\left(\tilde{T}>t\right))$, with $\hat{S}\left(t\right)$ as its estimator, the corresponding pseudo‐observations can be computed as

(9) $$\begin{array}{c} {\hat{S}}_{i}\left(t\right)=n\hat{S}\left(t\right)-\left(n-1\right){\hat{S}}_{\left(-i\right)}\left(t\right),\;i=1,\text{…},n, \end{array}$$

where ${\hat{S}}_{\left(-i\right)}\left(\cdot \right)$ denotes the survival estimator computed without the ith observation. In the right censoring case, $\hat{S}\left(\cdot \right)$ corresponds to the Kaplan–Meier estimator, and the values ${\hat{S}}_{i}\left(t\right)$ represent pseudo‐observations for the indicator function $I(\tilde{T}>t)$ at each time point t. For competing risks, $\hat{S}\left(\cdot \right)$ would be replaced by the Aalen–Johansen estimator (Aalen and Johansen 1978). However, under LBRC data, the traditional Kaplan–Meier estimator performs poorly because failing to account for left truncation can lead to substantial bias and reduce efficiency in estimating the survival distribution. In this setting, we consider two types of pseudo‐observations: one based on Vardi's NPMLE and another based on the estimator proposed by Wang et al. (2017). Because of the complexity of Vardi's estimator, it is hard to study the asymptotic properties of the regression estimator derived from it. Therefore, in Section 4, we focus on the asymptotic properties of jackknife pseudo‐observations derived from Wang's estimator. Nevertheless, in the simulation study, we compare pseudo‐observation approaches based on both estimators with another existing method within this framework.

## 3 Length‐Biased Cox Proportional Hazards Model

In this section, we investigate the effects of covariate on lifetime using the pseudo‐observation approach. To illustrate the estimation method, we assume that the unbiased lifetime $\tilde{T}$ satisfies a Cox PH model (Cox 1972). Throughout, we restrict attention to covariate‐independent censoring. Let the hazard function of $\tilde{T}$ given $\tilde{X}=x$ be denoted by λ(t|x). We assume that the target population follows the proportional hazards model as follows:

(10) $$\begin{array}{c} \lambda \left(t|x\right)=\lambda_{0}\left(t\right)\exp\left(x^{⊺}\beta \right), \end{array}$$

where $\lambda_{0}\left(t\right)$ is an unspecified baseline hazard function and β is a p×1 vector of unknown regression coefficients associated with covariates x. The cumulative baseline hazard function is $\Lambda_{0}\left(t\right)=\int_{0}^{t}\lambda_{0}\left(u\right)du$. It is important to note that the proportional hazards model in (10) may not hold for the observed (biased) population with survival time T. Consequently, several methods have been proposed for estimating the regression coefficients β under the LBRC setting. Notable contributions include those by Huang and Qin (2012), Huang et al. (2012), Liu et al. (2016), Qin and Shen (2010), Qin et al. (2011), Shen (2009), Tsai (2009), Wang (1996), and Wang and Zhou (2018). These methods are based on likelihood‐based and weighted estimating equations approaches. In contrast, we investigate inference for the Cox PH model using a pseudo‐observation approach as an alternative.

The expressions and representations in this section are based on conditional distributions, in contrast to the unconditional distributions considered earlier in Section 2. Let S(t|x) denote the covariate‐specific survival function of $\tilde{T}$. Under the Cox PH model in (10), it is straightforward to see that

(11) $$\begin{array}{c} \log\left(-\log S\left(t|x_{i}\right)\right)=\log\Lambda_{0}\left(t\right)+{x_{i}}^{⊺}\beta . \end{array}$$

Based on the survival estimators proposed by Vardi (1982b) and Wang et al. (2017), the corresponding survival pseudo‐observations can be constructed, respectively, as

(12) $$\begin{array}{c} {\hat{S}}_{i}^{V}\left(t\right)=n{\hat{S}}^{V}\left(t\right)-\left(n-1\right){\hat{S}}_{\left(-i\right)}^{V}\left(t\right),\;i=1,\text{…},n, \end{array}$$

and

(13) $$\begin{array}{c} {\hat{S}}_{i}^{W}\left(t\right)=n{\hat{S}}^{W}\left(t\right)-\left(n-1\right){\hat{S}}_{\left(-i\right)}^{W}\left(t\right),\;i=1,\text{…},n, \end{array}$$

where ${\hat{S}}^{V}\left(\cdot \right)$ and ${\hat{S}}^{W}\left(\cdot \right)$ denote the estimators defined in (2) and (5), respectively, and ${\hat{S}}_{\left(-i\right)}^{V}\left(\cdot \right)$ and ${\hat{S}}_{\left(-i\right)}^{W}\left(\cdot \right)$ are their corresponding leave‐one‐out estimates. As suggested by Klein and Andersen (2005), pseudo‐observations are typically calculated on a finite grid of time points $\tau_{1}<\cdots <\tau_{m}$.

Motivated by the work of Andersen et al. (2003), we use the survival pseudo‐observations in (12) and (13) as the response variables for fitting the Cox PH regression model in (11). In the subsequent analysis, we focus on the pseudo‐observations derived from the survival estimator proposed by Wang et al., ${\hat{S}}^{W}\left(t\right)$, as this is the key estimator under theoretical investigation. The pseudo‐observations based on Vardi's NPMLE, ${\hat{S}}^{V}\left(t\right)$, are included for comparison in the simulation study but they are not analyzed theoretically in depth. Based on $E\left({\hat{S}}_{i}^{W}\left(t\right)|X_{i}=x_{i}\right)=S\left(t|x_{i}\right)+o_{P}\left(1\right)$ , which will be proved in Lemma 4.3, we propose the following generalized linear model for the pseudo‐observation responses ${\hat{S}}_{i}^{W}\left(t\right)$ at a fixed time point t

(14) $$\begin{array}{c} \log\left(-\log E\left({\hat{S}}_{i}^{W}\left(t\right)|X_{i}=x_{i}\right)\right)=\log\Lambda_{0}\left(t\right)+x_{i}^{⊺}\beta ={\overset{ˇ}{x}}_{i}^{⊤}\theta , \end{array}$$

where we define $\beta_{0}=\log\Lambda_{0}\left(t\right)$ as the intercept in the regression model, and $\theta =(\beta_{0},\beta )$ denotes the full vector of regression coefficients. The covariate vector ${\overset{ˇ}{x}}_{i}$ includes a column of 1s appended to the covariate matrix $x_{i}$ to account for the intercept term. When analyzing multiple time points $\tau_{j}$, j=1,…,m, the model includes distinct intercepts $\beta_{0}=\left(\log\Lambda_{0}\left(\tau_{1}\right),\text{…},\log\Lambda_{0}\left(\tau_{m}\right)\right)$ for the time points $\tau_{j}$, j=1,…,m, and the full parameter vector becomes $\theta =(\beta_{0},\beta )$. The regression model in (14) can be estimated using standard GEE with the complementary log–log link function, defined as g(x)=log(−log(x)). Accordingly, the regression coefficients θ in model (14) can be estimated by solving the following estimating equation:

(15) $$U\left(\theta \right)\;=\;\sum_{i=1}^{n}U_{i}\left(\theta \right)\;=\;\sum_{i=1}^{n}{\left(\frac{\partial }{\partial \theta }g^{-1}\left({\overset{ˇ}{x}}_{i}^{⊤}\theta \right)\right)}^{⊤}V_{i}^{-1}\left({\hat{S}}_{i}^{W}\left(t\right)-g^{-1}\left({\overset{ˇ}{x}}_{i}^{⊤}\theta \right)\right)\;=\;0,$$

where $V_{i}$ is a working covariance matrix for the pseudo‐observations $\left({\hat{S}}_{i}^{W}\left(\tau_{1}\right),\text{…},{\hat{S}}_{i}^{W}\left(\tau_{m}\right)\right)$. In the case of a single time point, we set $V_{i}=1$.

## 4 Asymptotic Properties

In this section, we study the asymptotic properties of the estimators defined implicitly as solutions to the estimating equation in (15) under covariate‐independent censoring. The following lemma shows that, for large samples, the conditional expectation $E({\hat{S}}_{i}^{W}\left(t\right)|X_{i}=x_{i})$ closely approximates the true conditional survival function $S(t|x_{i})$. This property enables us to use the pseudo‐observations as outcome variables in the generalized linear model for assessing covariate effects.

To establish the large‐sample properties of the estimator obtained from (15), we adopt the GEE approach of Liang and Zeger (1986). For the theoretical results to hold, we assume the following regularity conditions:

a. The residual censoring time C is independent of (T,A), conditional on the covariates X.
b. For $0<t<u_{F}$, the integral $\int_{0}^{t}\frac{H(du)}{R(u)}$ is finite.

Remark 4.1 Assumption (a.) is reasonable because X represents baseline covariates, and censoring typically occurs after recruitment. Assumption (b.) ensures the validity of the asymptotic results in Theorem 4.4 under the pseudo‐observation framework. It is equivalent to requiring that the cumulative hazard function to be finite for $0<t<u_{F}$.

Remark 4.2 Depending on the specific statistical model used in the pseudo‐observation approach, different choices for the transformation function ψ(·) and link function g(·) can be considered. For example, in a generalized linear model, g(·) could be the log or identity link function. In the Cox PH model at a fixed time point, we take $\psi \left(\tilde{T}\right)=I\left(\tilde{T}\geq t\right)$ and g(x)=log(−log(1−x)). In competing risks settings, when the parameter of interest is the cause‐specific cumulative incidence function, $\psi \left(\tilde{T}\right)=I\left(\tilde{T}\leq t,D_{i}=d\right)$, where $D_{i}$ denotes the cause of death for subject i. For the τ‐mean restricted function, one may take $\psi \left(\tilde{T}\right)=\min\left(\tilde{T},\tau \right)$.

Lemma 4.3 Under conditions (a) and (b), we derive

$$\begin{array}{c} E\left({\hat{S}}_{i}^{W}\left(t\right)|X=x\right)=S\left(t|x\right)+o_{p}\left(1\right),\;a.s. \end{array}$$

See the Appendix.□

Theorem 4.4 Under conditions (a) and (b), the estimator $\hat{\theta }$, defined as the root of (15), is a consistent estimator of the true parameter vector θ. Moreover, $\hat{\theta }$ is asymptotically normal with mean zero and a covariance matrix that can be consistently estimated using the standard sandwich estimator $\hat{\Sigma }=I^{-1}\left(\hat{\theta }\right)\hat{Var}\left(U\left(\hat{\theta }\right)\right)I^{-1}\left(\hat{\theta }\right)$, where

$$\begin{array}{c} I\left(\hat{\theta }\right)=\sum_{i=1}^{n}{\left(\frac{\partial g^{-1}\left({\overset{ˇ}{x}}_{i}\hat{\theta }\right)}{\partial \theta }\right)}^{⊤}V_{i}^{-1}\left(\frac{\partial g^{-1}\left({\overset{ˇ}{x}}_{i}\hat{\theta }\right)}{\partial \theta }\right), \end{array}$$

and

$$\begin{array}{c} \hat{Var}\left(U\left(\hat{\theta }\right)\right)=\sum_{i=1}^{n}U_{i}{\left(\hat{\theta }\right)}^{⊤}U\left(\hat{\theta }\right), \end{array}$$

with $U_{i}\left(\cdot \right)$ as defined in (15).

See the Appendix.□

## 5 Simulation Study

In this section, we present Monte Carlo simulation studies to evaluate the finite‐sample performance of the proposed methods and compare them with existing approaches.

### 5.1 Simulation Setup

The unbiased survival time $\tilde{T}$ was generated from the Cox PH model of the form

$$\lambda \left(t|\tilde{X}\right)=\lambda_{0}\left(t\right)\exp\left(\beta_{1}{\tilde{X}}_{1}+\beta_{2}{\tilde{X}}_{2}\right),$$

where $\tilde{X}=\left({\tilde{X}}_{1},{\tilde{X}}_{2}\right)$, ${\tilde{X}}_{1}$ is a binary covariate taking values in {0,1} with an even distribution in the sample, and ${\tilde{X}}_{2}$ is a continuous covariate drawn from a uniform distribution on (−1,1). The true parameter values were set to $\left(\beta_{1},\beta_{2}\right)=\left(\log\left(2\right),\log\left(0.8\right)\right)$. For the baseline hazard function $\lambda_{0}\left(t\right)$, we considered three scenarios corresponding to Weibull distributions with different shape and scale parameters:

- **Scenario I:** $\lambda_{0}\left(t\right)=2$ (constant hazard)–Weibull with shape = 1, scale = 0.5.
- **Scenario II:** $\lambda_{0}\left(t\right)=2t$ (linear hazard)–Weibull with shape = 2, scale = 1.
- **Scenario III:** $\lambda_{0}\left(t\right)=24t^{2}$ (quadratic hazard)–Weibull with shape = 3, scale = 0.5.

The conditional survival function of $\tilde{T}$ given $\tilde{X}$, for example, under the constant hazard $\lambda_{0}\left(t\right)=2$, is given by

$$S\left(t|\tilde{X}\right)=\exp\left\{\left(-2t\right)\exp\left(\beta_{1}{\tilde{X}}_{1}+\beta_{2}{\tilde{X}}_{2}\right)\right\},$$

corresponding to a Weibull distribution with shape parameter 1 and scale parameter $0.5*\exp(-\beta_{1}{\tilde{X}}_{1}-\beta_{2}{\tilde{X}}_{2})$. To generate length‐biased data, we first drew ξ from a uniform distribution on (0,100) to mimic the incidence time of a stable disease. The enrollment time was set to 100, and the truncation time was defined as $\tilde{A}=100-\xi$. We kept only those pairs $\left(\tilde{A},\tilde{T}\right)$ such that $\tilde{A}\leq \tilde{T}$. Residual censoring time was generated independently from a uniform distribution on the interval (0,c), where c was selected to achieve approximate censoring rates of 10%,25%, and 50%. We reported the results for sample sizes n=250,500, and 1000. Each simulation scenario was replicated 2000 times to evaluate the finite‐sample properties of the proposed estimators under various conditions. Pseudo‐observations were calculated at 10 time points ranging from 0.4 to 1.3, with a spacing of 0.1.

We analyzed each scenario using the two proposed pseudo‐observations defined in Equations (12) and (13) to estimate parameters in the Cox PH model and the corresponding survival function. Within the pseudo‐observation framework, we compared the resulting estimators with a estimator proposed by Grand et al. (2019), designed for left‐truncated right‐censored data. Although the authors introduced two types of pseudo‐observations, both were shown to perform similarly; therefore, we only considered the first type in our simulations. These three pseudo‐observation approaches are referred to as “Vardi PO,” “Wang PO,” and “LTRC PO.” To further illustrate the performance of the pseudo‐observation methods, we included two standard estimators proposed by Qin and Shen (2010) and Huang and Qin (2012), denoted as “QS Cox” and “HQ Cox,” respectively. We also recorded the number of times the estimation procedures in the pseudo‐observation approach failed to produce valid estimates in each of the three scenarios. An invalid estimate was defined as a case where the GEE estimation procedure did not converge or produced an unrealistically extreme estimate.

For each scenario, we reported the empirical bias (Bias), calculated as the difference between the average estimate and the true parameter value; the empirical standard error (SE), computed as the standard deviation of the estimate; the root mean squared error (RMSE), obtained as the square root of the sum of the squared bias and squared SE, and the coverage probability (CP) of the 95% confidence intervals, calculated using the sandwich variance estimator for the pseudo‐observation methods and the model‐based standard errors for the standard methods.

### 5.2 Simulation Results

Tables 1, 2, 3 present the results of coefficient estimation for the Cox PH model under LBRC data, comparing the performance of five methods across three scenarios with varying sample sizes and censoring rates. All five estimators yield coefficient estimate that are close to the true values in all scenarios, indicating that they are asymptotically unbiased. Among the pseudo‐observation approaches, Vardi PO demonstrates greater efficiency compared to LTRC PO and Wang PO. The difference in SEs between Vardi PO and the likelihood‐based method HQ Cox ranges from 0.008 to 0.07 across all scenarios. The differences decrease as the sample size and censoring rate increase. Specifically, Scenario III (Table 3) shows smaller SE differences compared to Scenarios I and II. The number of invalid estimates in the pseudo‐observation approach decreases as the sample size increases. For a sample size of n=1000 with 50% censoring, the pseudo‐observation approach yielded the following results across 2000 replications: for the Vardi PO method, there were five, four, and eight cases of invalid regression estimates in Scenarios I, II, and III, respectively. For the Wang PO method, there were 11, 0, and 4 cases of invalid estimates in the same scenarios, while for the LTRC PO method, the counts were 18, 0, and 24.

**TABLE 1:** Simulation results for Cox PH model estimation under LBRC data with $\lambda_{0}\left(t\right)=2$.

			$\beta_{1}$				$\beta_{2}$			
n	C%	Method	Bias	SE	RMSE	CP	Bias	SE	RMSE	CP
250	10%	LTRC PO	−0.0267	0.2510	0.2524	0.9516	0.0415	0.2125	0.2165	0.9433
		Vardi PO	−0.0241	0.1826	0.1842	0.9544	0.0437	0.1248	0.1322	0.9123
		Wang PO	−0.0298	0.1928	0.1951	0.9487	0.0434	0.1370	0.1437	0.9215
		QS Cox	0.0166	0.1168	0.1180	0.9835	−0.0045	0.0885	0.0886	0.9855
		HQ Cox	0.0068	0.1125	0.1127	0.9305	−0.0014	0.0826	0.0826	0.9530
	25%	LTRC PO	−0.0262	0.2538	0.2551	0.9531	0.0399	0.2116	0.2153	0.9438
		Vardi PO	−0.0232	0.1850	0.1864	0.9533	0.0430	0.1273	0.1344	0.9082
		Wang PO	−0.0280	0.1998	0.2018	0.9452	0.0419	0.1413	0.1474	0.9273
		QS Cox	0.0151	0.1382	0.1390	0.9720	−0.0043	0.1095	0.1096	0.9760
		HQ Cox	0.0065	0.1209	0.1211	0.9335	−0.0018	0.0899	0.0899	0.9530
	50%	LTRC PO	−0.0226	0.2685	0.2694	0.9560	0.0376	0.2090	0.2124	0.9416
		Vardi PO	−0.0214	0.1927	0.1939	0.9533	0.0426	0.1331	0.1398	0.9168
		Wang PO	−0.0262	0.2066	0.2083	0.9538	0.0420	0.1527	0.1584	0.9291
		QS Cox	0.0108	0.1860	0.1863	0.9570	0.0003	0.1522	0.1522	0.9430
		HQ Cox	0.0059	0.1449	0.1450	0.9325	−0.0007	0.1140	0.1140	0.9340
500	10%	LTRC PO	−0.0324	0.1810	0.1839	0.9635	0.0457	0.1340	0.1416	0.9271
		Vardi PO	−0.0330	0.1238	0.1281	0.9570	0.0483	0.0889	0.1012	0.9033
		Wang PO	−0.0337	0.1419	0.1458	0.9605	0.0458	0.1549	0.1615	0.8997
		QS Cox	0.0080	0.0791	0.0795	0.9895	−0.0010	0.0625	0.0625	0.9895
		HQ Cox	0.0003	0.0761	0.0761	0.9450	0.0005	0.0583	0.0583	0.9560
	25%	LTRC PO	−0.0318	0.1831	0.1858	0.9610	0.0462	0.1356	0.1433	0.9276
		Vardi PO	−0.0328	0.1252	0.1294	0.9564	0.0489	0.0900	0.1024	0.8997
		Wang PO	−0.0330	0.1447	0.1484	0.9585	0.0464	0.1609	0.1675	0.9054
		QS Cox	0.0063	0.0926	0.0928	0.9795	0.0003	0.0764	0.0764	0.9730
		HQ Cox	0.0000	0.0813	0.0813	0.9425	0.0011	0.0632	0.0632	0.9535
	50%	LTRC PO	−0.0320	0.1899	0.1926	0.9615	0.0472	0.1416	0.1493	0.9341
		Vardi PO	−0.0317	0.1293	0.1331	0.9559	0.0497	0.0931	0.1055	0.9013
		Wang PO	−0.0316	0.1496	0.1529	0.9615	0.0481	0.1564	0.1636	0.9139
		QS Cox	0.0038	0.1264	0.1265	0.9585	0.0018	0.1015	0.1015	0.9605
		HQ Cox	−0.0006	0.0985	0.0985	0.9475	0.0031	0.0763	0.0764	0.9485
1000	10%	LTRC PO	−0.0428	0.1240	0.1312	0.9476	0.0446	0.0975	0.1072	0.9097
		Vardi PO	−0.0424	0.0971	0.1060	0.9384	0.0479	0.0673	0.0826	0.8482
		Wang PO	−0.0430	0.1028	0.1114	0.9417	0.0480	0.0736	0.0879	0.8658
		QS Cox	0.0059	0.0564	0.0567	0.9855	−0.0019	0.0443	0.0443	0.9900
		HQ Cox	0.0008	0.0553	0.0553	0.9315	−0.0005	0.0417	0.0417	0.9520
	25%	LTRC PO	−0.0423	0.1256	0.1325	0.9445	0.0452	0.0920	0.1025	0.9117
		Vardi PO	−0.0427	0.0979	0.1068	0.9359	0.0481	0.0681	0.0834	0.8552
		Wang PO	−0.0429	0.1043	0.1128	0.9427	0.0481	0.0749	0.0890	0.8688
		QS Cox	0.0059	0.0662	0.0665	0.9795	−0.0013	0.0527	0.0527	0.9785
		HQ Cox	0.0018	0.0591	0.0591	0.9365	−0.0009	0.0452	0.0452	0.9470
	50%	LTRC PO	−0.0432	0.1304	0.1374	0.9465	0.0435	0.0956	0.1050	0.9218
		Vardi PO	−0.0427	0.1002	0.1089	0.9388	0.0475	0.0696	0.0843	0.8596
		Wang PO	−0.0422	0.1089	0.1168	0.9422	0.0473	0.0769	0.0903	0.8813
		QS Cox	0.0032	0.0920	0.0921	0.9560	−0.0015	0.0711	0.0711	0.9630
		HQ Cox	0.0008	0.0709	0.0709	0.9335	−0.0009	0.0538	0.0538	0.9495

**TABLE 2:** Simulation results for Cox PH model estimation under LBRC data with $\lambda_{0}\left(t\right)=2t$.

			$\beta_{1}$				$\beta_{2}$			
n	C%	Method	Bias	SE	RMSE	CP	Bias	SE	RMSE	CP
250	10%	LTRC PO	0.0042	0.2233	0.2233	0.9555	−0.0011	0.1871	0.1871	0.9590
		Vardi PO	0.0024	0.1885	0.1885	0.9560	0.0000	0.1610	0.1610	0.9555
		Wang PO	0.0019	0.1920	0.1920	0.9545	0.0006	0.1625	0.1625	0.9530
		QS Cox	0.0183	0.1291	0.1304	0.9670	−0.0044	0.1036	0.1037	0.9655
		HQ Cox	0.0126	0.1260	0.1266	0.8920	−0.0023	0.1004	0.1004	0.9040
	25%	LTRC PO	0.0045	0.2296	0.2296	0.9570	−0.0022	0.1950	0.1950	0.9555
		Vardi PO	0.0024	0.1916	0.1916	0.9575	−0.0005	0.1657	0.1657	0.9535
		Wang PO	0.0015	0.1961	0.1961	0.9555	0.0000	0.1692	0.1692	0.9515
		QS Cox	0.0215	0.1482	0.1498	0.9635	−0.0033	0.1203	0.1203	0.9585
		HQ Cox	0.0130	0.1358	0.1364	0.8905	−0.0019	0.1089	0.1089	0.9025
	50%	LTRC PO	0.0124	0.2511	0.2514	0.9580	−0.0055	0.2146	0.2147	0.9505
		Vardi PO	0.0075	0.2041	0.2042	0.9549	−0.0026	0.1749	0.1749	0.9544
		Wang PO	0.0069	0.2135	0.2136	0.9625	−0.0042	0.1819	0.1819	0.9585
		QS Cox	0.0168	0.1965	0.1972	0.9360	−0.0058	0.1624	0.1625	0.9400
		HQ Cox	0.0147	0.1612	0.1619	0.9060	−0.0035	0.1301	0.1301	0.9120
500	10%	LTRC PO	−0.0044	0.1521	0.1522	0.9635	0.0046	0.1342	0.1343	0.9530
		Vardi PO	−0.0090	0.1270	0.1273	0.9630	0.0044	0.1138	0.1139	0.9540
		Wang PO	−0.0093	0.1300	0.1303	0.9585	0.0043	0.1163	0.1164	0.9510
		QS Cox	0.0104	0.0857	0.0863	0.9770	0.0005	0.0695	0.0695	0.9790
		HQ Cox	0.0057	0.0837	0.0839	0.9105	0.0016	0.0688	0.0688	0.9065
	25%	LTRC PO	−0.0029	0.1562	0.1562	0.9635	0.0053	0.1387	0.1388	0.9520
		Vardi PO	−0.0081	0.1320	0.1322	0.9635	0.0048	0.1171	0.1172	0.9510
		Wang PO	−0.0081	0.1339	0.1341	0.9605	0.0055	0.1205	0.1206	0.9505
		QS Cox	0.0123	0.1018	0.1025	0.9625	0.0013	0.0810	0.0810	0.9695
		HQ Cox	0.0073	0.0912	0.0915	0.9175	0.0029	0.0735	0.0736	0.9130
	50%	LTRC PO	−0.0038	0.1708	0.1708	0.9635	0.0034	0.1488	0.1488	0.9550
		Vardi PO	−0.0087	0.1378	0.1381	0.9650	0.0038	0.1225	0.1226	0.9500
		Wang PO	−0.0083	0.1449	0.1451	0.9610	0.0037	0.1285	0.1286	0.9565
		QS Cox	0.0073	0.1366	0.1368	0.9375	−0.0008	0.1090	0.1090	0.9515
		HQ Cox	0.0049	0.1089	0.1090	0.9175	0.0016	0.0862	0.0862	0.9280
1000	10%	LTRC PO	−0.0114	0.1053	0.1059	0.9510	0.0034	0.0924	0.0925	0.9580
		Vardi PO	−0.0149	0.0891	0.0903	0.9585	0.0041	0.0815	0.0816	0.9575
		Wang PO	−0.0152	0.0895	0.0908	0.9560	0.0037	0.0822	0.0823	0.9565
		QS Cox	0.0067	0.0613	0.0617	0.9730	−0.0026	0.0514	0.0515	0.9720
		HQ Cox	0.0036	0.0606	0.0607	0.8965	−0.0014	0.0501	0.0501	0.8895
	25%	LTRC PO	−0.0104	0.1074	0.1079	0.9575	0.0034	0.0958	0.0959	0.9560
		Vardi PO	−0.0151	0.0954	0.0966	0.9555	0.0045	0.0858	0.0859	0.9530
		Wang PO	−0.0145	0.0916	0.0927	0.9560	0.0040	0.0852	0.0853	0.9530
		QS Cox	0.0074	0.0717	0.0721	0.9590	−0.0014	0.0584	0.0584	0.9670
		HQ Cox	0.0041	0.0653	0.0654	0.9035	−0.0007	0.0540	0.0540	0.8945
	50%	LTRC PO	−0.0094	0.1172	0.1176	0.9560	0.0046	0.1056	0.1057	0.9530
		Vardi PO	−0.0141	0.0940	0.0951	0.9584	0.0041	0.0877	0.0878	0.9554
		Wang PO	−0.0139	0.0996	0.1006	0.9625	0.0037	0.0926	0.0927	0.9510
		QS Cox	0.0039	0.0945	0.0946	0.9520	−0.0020	0.0763	0.0763	0.9535
		HQ Cox	0.0044	0.0778	0.0779	0.9090	0.0001	0.0631	0.0631	0.9125

**TABLE 3:** Simulation results for Cox PH model estimation under LBRC data with $\lambda_{0}\left(t\right)=24t^{2}$.

			$\beta_{1}$				$\beta_{2}$			
n	C%	Method	Bias	SE	RMSE	CP	Bias	SE	RMSE	CP
250	10%	LTRC PO	0.0272	0.1828	0.1848	0.9576	0.0068	0.1461	0.1463	0.9471
		Vardi PO	0.0244	0.1596	0.1615	0.9517	0.0092	0.1172	0.1176	0.9558
		Wang PO	0.0243	0.1628	0.1646	0.9561	0.0092	0.1220	0.1223	0.9545
		QS Cox	0.0176	0.1344	0.1355	0.9610	−0.0057	0.1056	0.1058	0.9685
		HQ Cox	0.0138	0.1322	0.1329	0.8705	−0.0037	0.1054	0.1055	0.8850
	25%	LTRC PO	0.0339	0.1997	0.2026	0.9621	0.0027	0.1599	0.1599	0.9499
		Vardi PO	0.0286	0.1700	0.1724	0.9504	0.0065	0.1265	0.1267	0.9563
		Wang PO	0.0288	0.1752	0.1776	0.9597	0.0042	0.1324	0.1325	0.9516
		QS Cox	0.0196	0.1500	0.1513	0.9540	−0.0091	0.1210	0.1213	0.9605
		HQ Cox	0.0137	0.1413	0.1420	0.8715	−0.0061	0.1136	0.1138	0.8835
	50%	LTRC PO	0.0530	0.2475	0.2531	0.9676	−0.0073	0.2036	0.2037	0.9496
		Vardi PO	0.0370	0.2000	0.2034	0.9592	0.0043	0.1531	0.1532	0.9517
		Wang PO	0.0398	0.2151	0.2188	0.9686	0.0031	0.1637	0.1637	0.9565
		QS Cox	0.0128	0.1961	0.1965	0.9390	−0.0097	0.1610	0.1613	0.9445
		HQ Cox	0.0133	0.1682	0.1687	0.8955	−0.0085	0.1370	0.1373	0.8955
500	10%	LTRC PO	0.0186	0.1230	0.1244	0.9614	0.0141	0.1048	0.1057	0.9384
		Vardi PO	0.0145	0.1072	0.1082	0.9559	0.0137	0.0887	0.0898	0.9404
		Wang PO	0.0144	0.1087	0.1096	0.9579	0.0135	0.0909	0.0919	0.9364
		QS Cox	0.0080	0.0897	0.0901	0.9725	−0.0026	0.0756	0.0756	0.9640
		HQ Cox	0.0050	0.0892	0.0893	0.8830	−0.0015	0.0752	0.0752	0.8830
	25%	LTRC PO	0.0208	0.1335	0.1351	0.9616	0.0141	0.1149	0.1158	0.9339
		Vardi PO	0.0164	0.1141	0.1153	0.9578	0.0136	0.0951	0.0961	0.9387
		Wang PO	0.0164	0.1169	0.1180	0.9593	0.0141	0.0987	0.0997	0.9312
		QS Cox	0.0106	0.1006	0.1012	0.9675	−0.0013	0.0860	0.0860	0.9550
		HQ Cox	0.0060	0.0954	0.0956	0.8985	−0.0005	0.0815	0.0815	0.8830
	50%	LTRC PO	0.0276	0.1707	0.1729	0.9664	0.0169	0.1412	0.1422	0.9401
		Vardi PO	0.0202	0.1377	0.1392	0.9510	0.0133	0.1100	0.1108	0.9410
		Wang PO	0.0234	0.1448	0.1467	0.9639	0.0141	0.1181	0.1189	0.9352
		QS Cox	0.0095	0.1322	0.1325	0.9470	−0.0053	0.1163	0.1164	0.9320
		HQ Cox	0.0079	0.1141	0.1144	0.8990	−0.0011	0.0984	0.0984	0.8800
1000	10%	LTRC PO	0.0065	0.0871	0.0873	0.9610	0.0138	0.0710	0.0723	0.9470
		Vardi PO	0.0041	0.0767	0.0768	0.9550	0.0157	0.0605	0.0625	0.9370
		Wang PO	0.0048	0.0780	0.0781	0.9515	0.0157	0.0619	0.0639	0.9400
		QS Cox	0.0045	0.0642	0.0644	0.9645	−0.0001	0.0528	0.0528	0.9695
		HQ Cox	0.0018	0.0645	0.0645	0.8770	0.0004	0.0526	0.0526	0.8755
	25%	LTRC PO	0.0072	0.0932	0.0935	0.9620	0.0124	0.0765	0.0775	0.9490
		Vardi PO	0.0044	0.0818	0.0819	0.9614	0.0149	0.0648	0.0665	0.9379
		Wang PO	0.0058	0.0837	0.0839	0.9585	0.0147	0.0669	0.0685	0.9355
		QS Cox	0.0071	0.0719	0.0722	0.9635	−0.0012	0.0599	0.0599	0.9630
		HQ Cox	0.0027	0.0689	0.0690	0.8890	−0.0004	0.0566	0.0566	0.8805
	50%	LTRC PO	0.0082	0.1172	0.1175	0.9585	0.0146	0.0940	0.0951	0.9448
		Vardi PO	0.0055	0.0963	0.0965	0.9513	0.0169	0.0749	0.0768	0.9448
		Wang PO	0.0080	0.1013	0.1016	0.9579	0.0173	0.0801	0.0819	0.9414
		QS Cox	0.0058	0.0944	0.0946	0.9575	0.0029	0.0799	0.0800	0.9385
		HQ Cox	0.0037	0.0824	0.0825	0.8925	0.0016	0.0672	0.0672	0.9030

**FIGURE 1:**
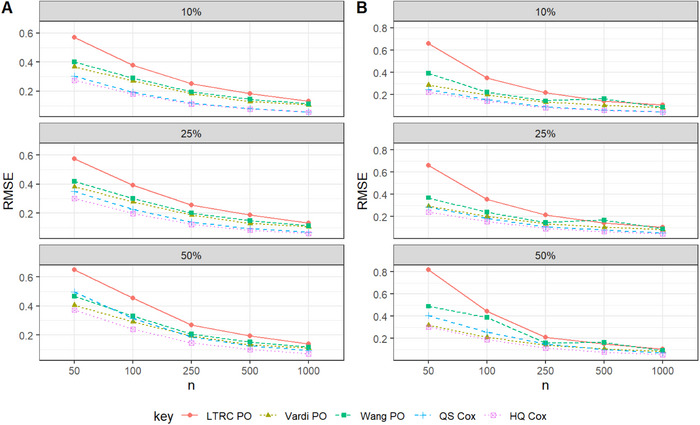
RMSE performance in Scenario I with $\lambda_{0}\left(t\right)=2$: Effect of sample size and censoring rate on the estimates of $\beta_{1}$ (Panel A) and $\beta_{2}$ (Panel B).

**FIGURE 2:**
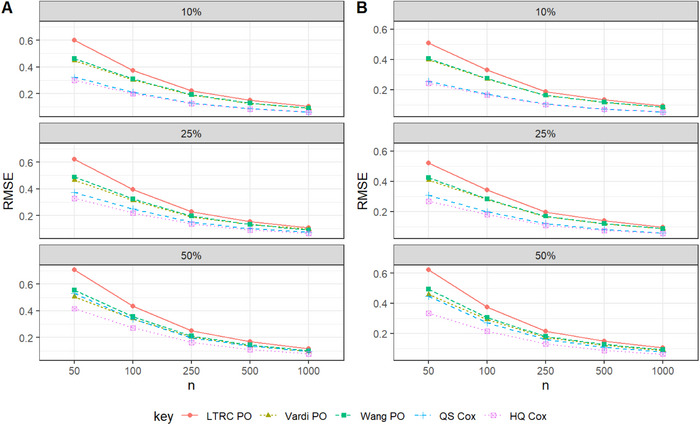
RMSE performance in Scenario II with $\lambda_{0}\left(t\right)=2t$: Effect of sample size and censoring rate on the estimates of $\beta_{1}$ (Panel A) and $\beta_{2}$ (Panel B).

**FIGURE 3:**
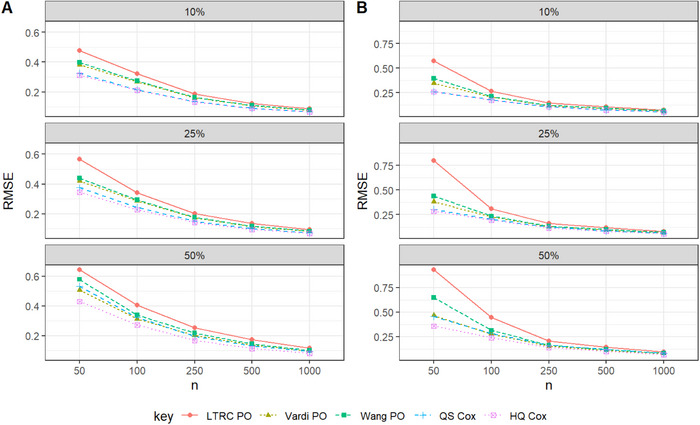
RMSE performance in Scenario III with $\lambda_{0}\left(t\right)=24t^{2}$: Effect of sample size and censoring rate on the estimates of $\beta_{1}$ (Panel A) and $\beta_{2}$ (Panel B).

Overall, we found that the two proposed pseudo‐observation approaches perform comparably to the HQ Cox method in terms of SE in large samples. The CPs reported in Tables 1–3 approximately match the nominal 95% confidence level. An exception is observed for continuous covariates in Scenario I, where CPs for the pseudo‐observation methods fall slightly below 95%, while those for the QS Cox method tends to exceed 95%. Although HQ Cox provides lower SEs in Tables 2 and 3, its CPs deviate from the nominal level. In these two scenarios, a slight increase in the standard error of the two proposed pseudo‐observation‐based estimators can result in improved coverage probabilities, particularly with larger sample sizes. Table 4 presents the simulation results for survival estimation under LBRC data across all scenarios with a sample size of 500. It can be observed that the Vardi PO method shows lower relative efficiency (RE) compared to HQ Cox at larger evaluation times (e.g., t=1.6 and 2), indicating improved survival prediction performance in the tails of the underlying distribution. Figures 1, 2, 3 visualize the RMSE values for the pseudo‐observation approaches and standard methods used to estimate Cox regression coefficients, considering different censoring scenarios and sample sizes of n=50,100,250,500, and 1000.

**TABLE 4:** Simulation results for survival estimation under LBRC data across all scenarios with a sample size of 500.

		$\lambda_{0}\left(t\right)=2$					$\lambda_{0}\left(t\right)=2t$					$\lambda_{0}\left(t\right)=24t^{2}$				
		HQ Cox		Vardi PO			HQ Cox		Vardi PO			HQ Cox		Vardi PO		
C%	time	Bias	SE	Bias	SE	RE	Bias	SE	Bias	SE	RE	Bias	SE	Bias	SE	RE
10%	0.5	−0.0777	0.0348	−0.1329	0.0344	2.5986	−0.0713	0.0279	−0.0877	0.0309	1.4757	−0.2299	0.0285	−0.2469	0.0284	1.1509
	0.8	−0.2442	0.0244	−0.2871	0.0206	1.3756	−0.3216	0.0303	−0.3493	0.0344	1.1806	−0.5814	0.0063	−0.5843	0.0061	1.0100
	1.6	−0.4076	0.0079	−0.3934	0.0094	0.9317	−0.7732	0.0132	−0.6877	0.0200	0.7915	−0.5983	0.0004	−0.5843	0.0061	0.9539
	2.0	−0.4301	0.0046	−0.3934	0.0094	0.8370	−0.8326	0.0060	−0.6877	0.0200	0.6828	−0.5983	0.0004	−0.5843	0.0061	0.9539
25%	0.5	0.0036	0.0358	−0.0515	0.0347	2.9705	0.0024	0.0288	−0.0143	0.0323	1.5017	0.0017	0.0303	−0.0155	0.0300	1.2335
	0.8	−0.1627	0.0256	−0.2056	0.0209	1.5745	−0.2477	0.0320	−0.2757	0.0364	1.2398	−0.3495	0.0073	−0.3529	0.0071	1.0195
	1.6	−0.3258	0.0090	−0.3120	0.0099	0.9173	−0.6992	0.0145	−0.6143	0.0214	0.7725	−0.3661	0.0009	−0.3529	0.0071	0.9296
	2.0	−0.3482	0.0057	−0.3120	0.0099	0.8035	−0.7587	0.0071	−0.6143	0.0214	0.6563	−0.3661	0.0009	−0.3529	0.0071	0.9296
50%	0.5	0.0708	0.0396	0.0153	0.0357	0.2290	0.0837	0.0309	0.0671	0.0338	0.7095	0.1932	0.0365	0.1750	0.0351	0.8240
	0.8	−0.0951	0.0299	−0.1389	0.0224	1.9914	−0.1667	0.0369	−0.1948	0.0391	1.3543	−0.1571	0.0102	−0.1619	0.0092	1.0610
	1.6	−0.2574	0.0125	−0.2454	0.0118	0.9089	−0.6170	0.0186	−0.5336	0.0248	0.7489	−0.1729	0.0030	−0.1619	0.0092	0.8794
	2.0	−0.2794	0.0084	−0.2454	0.0118	0.7725	−0.6754	0.0098	−0.5336	0.0248	0.6254	−0.1729	0.0030	−0.1619	0.0092	0.8794

Based on the simulation results presented in Tables 1–3, we conclude the following:

- Across all settings, the point estimates of the proposed methods appear to be unbiased.
- Among the pseudo‐observation approaches, both proposed estimators, Vardi PO and Wang PO, perform better in terms of RMSE compared to LTRC PO; however, Vardi PO consistently yields the lowest RMSE within the pseudo‐observation approaches, as expected.
- Across all settings, the pseudo‐observation approaches are comparable to the likelihood‐based method HQ Cox, which possesses the lowest SE. Specifically, when the baseline hazard function follows a thin‐tailed distribution (Scenarios II and III), the SEs are closer. In these scenarios, although HQ Cox provides lower SEs, its CPs deviate from the nominal level. This issue affects the validity and reliability of the HQ Cox estimator. Therefore, in these two scenarios, accepting a slight increase in SE can lead to improved coverage probabilities, especially for larger sample sizes.
- The suggested estimation methods perform well for LBRC data in terms of the CP approximately matching the nominal 95% confidence level.
- For a fix sample size, as the censoring rate increases in the simulation scenarios, the Bias, SE, and RMSE of the estimates also increases.

## 6 Channing House Data

In this section, we analyze the Channing House data (Hyde 1980) using the proposed methods. This dataset includes 462 retirees (97 men and 365 women) who participated in the study from January 1964 to July 1975. To align with the length‐biased framework, we focused on individuals aged 65 years or older as the target group of interest. A total of 450 individuals were included in this subset. We assessed the stationarity assumption using the method suggested by Addona and Wolfson (2006), implemented in the CoxPhLb package in R. The results indicated that the subset of individuals aged 65 or older at entry satisfies the length‐biased assumption (*p*‐value = 0.794). According to the records, 44 men and 128 women died at Channing House, resulting in a censoring rate of 62%. To investigate whether gender affects the survival risk of retirees in the length‐biased subset, we applied a Cox PH model to assess the association between gender and survival times. The covariate is a binary variable, with X=1 indicating a male subject (reference group). For the pseudo‐observation approach, we used 9 quantiles of the uncensored observations, ranging from the 10th to the 90th percentile, as the time points for calculating the pseudo‐observations. Table 5 shows the results from different pseudo‐observation methods used to estimate the coefficient in the Cox PH model. The table reports the hazard ratio (HR) for females, representing their relative risk of death compared to males. The HRs obtained from our two proposed methods are 0.9095 and 0.8322, while the HR from the LTRC PO method is 0.7441. However, none of these hazard ratios are statistically significant (p-value>0.05), suggesting that gender does not have a significant effect on the survival of retirees in the length‐biased subset. Notably, these findings are consistent with those reported in Table 3.7 of Akbari et al. (2024), which presents results based on standard methods.

**TABLE 5:** Cox PH model results using different estimating equations for the Channing House data.

Method	Coef(Female)	Exp(coef)	SE	*p*‐value	95% CI
LTRC PO	−0.2956	0.7441	0.2329	0.2045	(−0.7521, 0.1610)
Vardi PO	−0.0948	0.9095	0.1994	0.6345	(−0.4857, 0.2961)
Wang PO	−0.1837	0.8322	0.2035	0.3667	(−0.5826, 0.2151)

## 7 Concluding Remarks

Length‐biased sampling induces bias into the observed survival times, making it important to account for this in the analysis. Despite standard methods, which usually provides efficient estimators, alternative approaches may offer advantages in some situations. Grand et al. (2019) proposed two types of pseudo‐observations under left‐truncation right‐censored data, which can also be applied for LBRC data. However, under the stationarity assumption, some information may be lost when using the pseudo‐observations proposed by Grand et al. (2019) to estimate coefficients in the Cox PH model. Hence, we extended the pseudo‐observation approach based on nonparametric survival functions specifically designed for LBRC data to improve the precision of pseudo‐observations under such conditions. To provide a comprehensive comparison, we evaluated the performance of pseudo‐observation‐based estimators against two prominent standard methods. Simulation studies demonstrated that, within the pseudo‐observation framework, the proposed estimators outperformed the method introduced by Grand et al. (2019) under LBRC data. Comparing with the standard methods, we found no substantial differences, and our approaches yielded comparable SEs, particularly in large sample sizes. An important advantage of our approach is its applicability in complex situations where standard methods might not be feasible. For example, in estimating Cox regression models with time‐varying covariates under LBRC data, an area motivated by Whata et al. (2024), the pseudo survival probabilities could be used to build the neural networks introduced by Zhao and Feng (2020) and Feng and Zhao (2021). From a theoretical perspective, we showed that, under certain regularity conditions, the root of the GEE derived from the pseudo‐observations based on the nonparametric survival estimator proposed by Wang et al. (2017) is consistent and asymptotically normally distributed.

As future work, one can extend the pseudo‐observation approach to settings with LBRC data and competing risks, which are common. In this case, one could focus on the cause‐specific cumulative incidence function to obtain estimates of relative risks. Additionally, the precision of these proposed nonparametric pseudo‐observation approaches could be increased by using parametric pseudo‐observations, combining flexible parametric models with pseudo‐observations, as suggested by Johansen et al. (2020). However, the validity of parametric pseudo‐observations depends on the fulfillment of certain assumptions. Specifically, this approach requires the assumption of independent censoring, as well as the appropriate choice of the number and positions of knots for the splines, as discussed in Johansen et al. (2021).

## Conflicts of Interest

The authors declare that there is no conflict of interest.

## Open Research Badges

This article has earned an Open Data badge for making publicly available the digitally‐shareable data necessary to reproduce the reported results. The data is available in the Supporting Information section.

This article has earned an open data badge “**Reproducible Research**” for making publicly available the code necessary to reproduce the reported results. The results reported in this article could fully be reproduced.

## Supporting information

Supporting Information

## Data Availability

The data that support the findings of this study are available in "KMsurv" at https://rdrr.io/cran/KMsurv/man/channing.html. These data were derived from the following resources available in the public domain: ‐ R package, https://rdrr.io/cran/KMsurv/man/channing.html

## Appendix

This section first presents several preliminary lemmas that will be used in the proof of Proposition 2.1. To avoid the issue of taking the logarithm of zero, we introduce a slight modification to the product‐limit estimator ${\hat{S}}^{W}\left(\cdot \right)$. In the following lemma, we demonstrate that this modified estimator, denoted by ${\bar{S}}_{n}\left(\cdot \right)$, behaves in the same way as ${\hat{S}}^{W}\left(\cdot \right)$, where

(A1)
$$\begin{array}{c} {\bar{S}}_{n}\left(t\right)=\prod_{i:Y_{i}\leq t}\left(1-\frac{\Delta_{i}}{nR_{n}\left(Y_{i}\right)+1}\right). \end{array}$$

Lemma A.1 If $\int_{0}^{b}dH\left(u\right)/R^{2}\left(u\right)<\infty$, then uniformly for $0\leq t\leq b<\tau_{F}$, we have

$$\begin{array}{c} {\hat{S}}^{W}\left(t\right)-{\bar{S}}_{n}\left(t\right)=O_{p}\left(n^{-1}\right). \end{array}$$

According to the definition of ${\hat{S}}^{W}\left(t\right)$ in (5), we have

$$\begin{array}{c} {\hat{S}}^{W}\left(t\right)-{\bar{S}}_{n}\left(t\right)=\prod_{i:Y_{i}\leq t}\left(1-\frac{\Delta_{i}}{nR_{n}\left(Y_{i}\right)}\right)-\prod_{i:Y_{i}\leq t}\left(1-\frac{\Delta_{i}}{nR_{n}\left(Y_{i}\right)+1}\right). \end{array}$$

By applying the inequality $|\prod_{i=1}^{n}c_{i}-\prod_{i=1}^{n}d_{i}|\leq \sum_{i=1}^{n}\left|c_{i}-d_{i}\right|$, for $|c_{i}\left|,\right|d_{i}|\leq 1$ (Diehl and Stute 1988) and using the definition of the empirical subdistribution function $H_{n}\left(\cdot \right)$, we obtain

$$\begin{array}{cc} |{\hat{S}}^{W}\left(t\right)-{\bar{S}}_{n}\left(t\right)| & \leq \sum_{i:Y_{i}\leq t}\frac{\Delta_{i}}{n^{2}R_{n}^{2}\left(Y_{i}\right)}\\ & \leq n^{-1}\int_{0}^{b}\frac{1}{R_{n}^{2}\left(u\right)}dH_{n}\left(u\right)\\ & \leq n^{-1}\underset{0<u<b}{\sup}\frac{R^{2}\left(u\right)}{R_{n}^{2}\left(u\right)}\int_{0}^{b}\frac{1}{R^{2}\left(u\right)}dH_{n}\left(u\right). \end{array}$$

Note that $\sum_{j=1}^{n}R_{j}\left(Y_{i}\right)=\sum_{j=1}^{n}I\left(A_{j}\leq Y_{i}\leq Y_{j}\right)\geq 1$ for any i=1,…,n, which implies $|1-\frac{\Delta_{i}}{nR_{n}\left(Y_{i}\right)}|\leq 1$ and $|1-\frac{\Delta_{i}}{nR_{n}\left(Y_{i}\right)+1}|\leq 1$. By the strong law of large numbers (SLLN), as n→∞, the integral $\int_{0}^{b}\frac{1}{R^{2}\left(u\right)}dH_{n}\left(u\right)$ converges to $\int_{0}^{b}\frac{1}{R^{2}\left(u\right)}dH\left(u\right)$, which is finite by assumption. Furthermore, by the Dvoretzky–Kiefer–Wolfowitz (DKW) (Shorack and Wellner 2009) inequality for the empirical distribution function, we have

(A2)
$$\begin{array}{c} \underset{0<t<b}{\sup}\left|R_{n}\left(t\right)-R\left(t\right)\right|=O_{p}\left(n^{-1/2}\right). \end{array}$$

Consequently, as n→∞,

$$\begin{array}{cc} \underset{0<u<b}{\sup}\left|\frac{R^{2}\left(u\right)}{R_{n}^{2}\left(u\right)}\right| & \leq 1+\underset{0<u<b}{\sup}\frac{|R_{n}^{2}\left(u\right)-R^{2}\left(u\right)|}{R_{n}^{2}\left(u\right)}\\ & \leq 1+2\underset{0<u<b}{\sup}\frac{|R_{n}\left(u\right)-R\left(u\right)|}{R_{n}^{2}\left(u\right)}\\ & =1+o_{p}\left(1\right). \end{array}$$

Thus, the desired conclusion follows.□

Lemma A.2 For any $b<\tau_{F}$, we have

$$\begin{array}{c} \underset{0<t\leq b}{\sup}\left|\int_{0}^{t}\left(\frac{1}{R_{n}\left(u\right)}-\frac{1}{R(u)}\right)d\left[H_{n}\left(u\right)-H\left(u\right)\right]\right|=O\left(n^{-3/4}\log n\right)\;a.s. \end{array}$$

Divide the interval [0,b] into subintervals $\left[x_{i},x_{i+1}\right]$; $i=1,\text{…},\nu_{n}$, such that $\nu_{n}=O\left(n^{1/2}\right)$ and $0=x_{1}<x_{2}<\cdots <x_{\nu_{n}+1}=b$. Observe that

$$\begin{array}{cc} \underset{0\leq t\leq b}{\sup} & |\int_{0}^{t}\left(\frac{1}{R_{n}\left(u\right)}-\frac{1}{R(u)}\right)d\left[H_{n}\left(u\right)-H\left(u\right)\right]|\\ \leq & \max_{1\leq i\leq \nu_{n}}\sum_{j=1}^{i-1}\int_{x_{j}}^{x_{j+1}}\left|\left(\frac{1}{R_{n}\left(u\right)}-\frac{1}{R(u)}\right)d\left[H_{n}\left(u\right)-H\left(u\right)\right]\right|\\ & \cdot \max_{1\leq i\leq \nu_{n}}\underset{x_{i}\leq t\leq x_{i+1}}{\sup}\int_{x_{i}}^{t}\left|\left(\frac{1}{R_{n}\left(u\right)}-\frac{1}{R(u)}\right)d\left[H_{n}\left(u\right)-H\left(u\right)\right]\right|\\ =: & I+I\;I. \end{array}$$

Equation (A2) and the modulus of continuity of the empirical process imply that

(A3)
$$\begin{array}{cc} I & \leq \underset{0\leq t\leq b}{\sup}|\frac{1}{R_{n}\left(t\right)}-\frac{1}{R(t)}|\nu_{n}\max_{1\leq i\leq \nu_{n}}\left|H_{n}\left(x_{i+1}\right)-H\left(x_{i+1}\right)-H_{n}\left(x_{i}\right)+H\left(x_{i}\right)\right|\\ & =O\left(n^{-3/4}\log n\right)\;a.s. \end{array}$$

Next, for II, we have

$$\begin{array}{cc} I\;I\leq & \max_{1\leq i\leq \nu_{n}}\underset{x_{i}\leq t\leq x_{i+1}}{\sup}\int_{x_{i}}^{t}\left|\left(\;\;\frac{1}{R_{n}\left(u\right)}\;-\;\frac{1}{R(u)}\;-\;\frac{1}{R_{n}\left(x_{i}\right)}\;+\;\frac{1}{R(x_{i})}\;\;\right)d\left[H_{n}\left(u\right)-H\left(u\right)\right]\right|\\ & +\underset{0\leq t\leq b}{\sup}|\frac{1}{R_{n}\left(t\right)}-\frac{1}{R(t)}|\max_{1\leq i\leq \nu_{n}}\underset{x_{i}\leq t\leq x_{i+1}}{\sup}\left|H_{n}\left(t\right)-H\left(t\right)-H_{n}\left(x_{i}\right)+H\left(x_{i}\right)\right|\\ \leq & \;2\max_{1\leq i\leq \nu_{n}}\underset{x_{i}\leq t\leq x_{i+1}}{\sup}\left|\frac{1}{R_{n}\left(t\right)}-\frac{1}{R(t)}-\frac{1}{R_{n}\left(x_{i}\right)}+\frac{1}{R(x_{i})}\right|+O\left(n^{-1}\log n\right),\;a.s. \end{array}$$

Since $\sup_{0<t<b}{\left(R_{n}\left(t\right)-R\left(t\right)\right)}^{2}=O\left(n^{-1}\log\log n\right)$ a.s., we have

$$\begin{array}{cc} I\;I\;I & :=\max_{1\leq i\leq \nu_{n}}\underset{x_{i}\leq t\leq x_{i+1}}{\sup}\left|\frac{1}{R_{n}\left(t\right)}-\frac{1}{R(t)}-\frac{1}{R_{n}\left(x_{i}\right)}+\frac{1}{R(x_{i})}\right|\\ & \leq \max_{1\leq i\leq \nu_{n}}\underset{x_{i}\leq t\leq x_{i+1}}{\sup}\left|\frac{R_{n}\left(t\right)-R\left(t\right)}{R^{2}\left(t\right)}-\frac{R_{n}\left(x_{i}\right)-R\left(x_{i}\right)}{R^{2}\left(x_{i}\right)}\right|+O\left(n^{-1}\log\log n\right),\;a.s.\\ & \leq \underset{0\leq t\leq b}{\sup}|R_{n}\left(t\right)-R\left(t\right)|\max_{1\leq i\leq \nu_{n}}\underset{x_{i}\leq t\leq x_{i+1}}{\sup}\left|\frac{1}{R^{2}\left(t\right)}-\frac{1}{R^{2}\left(x_{i}\right)}\right|\\ & \;+\;\max_{1\leq i\leq \nu_{n}}\frac{1}{R^{2}\left(x_{i}\right)}\max_{1\leq i\leq \nu_{n}}\underset{x_{i}\leq t\leq x_{i+1}}{\sup}\left|R_{n}\left(t\right)-R\left(t\right)-R_{n}\left(x_{i}\right)+R\left(x_{i}\right)\right|\\ & \;+\;O(n^{-1}\log\log n),\;a.s. \end{array}$$

Here, $\max_{i}\left|x_{i+1}-x_{i}\right|=O\left(n^{-1/2}\right)$. An application of the modulus of continuity of the empirical process yields

$$\begin{array}{c} I\;I\;I=O\left(n^{-3/4}\log n\right),\;a.s. \end{array}$$

Moreover, we conclude

$$\begin{array}{c} I\;I=O\left(n^{-3/4}\log n\right),\;a.s., \end{array}$$

which together with (A3) yield the result.□

Lemma A.3 Let Λ(·) denote the cumulative hazard function of $\tilde{T}$, defined as $\int_{0}^{t}\frac{dH(u)}{R(u)}$, and let $\Lambda_{n}\left(\cdot \right)$ be a natural estimator of Λ(·). If $b<\tau_{F}$, then for 0≤t≤b, we have

$${\hat{\Lambda }}_{n}\left(t\right)-\Lambda \left(t\right)=n^{-1}\sum_{i=1}^{n}\phi_{i}\left(t\right)+L_{n}\left(t\right),$$

where

$$\underset{0\leq t\leq b}{\sup}\left|L_{n}\left(t\right)\right|=O\left(n^{-3/4}\log n\right)\;a.s.$$

By the definition $\Lambda_{n}\left(t\right)=\int_{0}^{t}\frac{dH_{n}\left(u\right)}{R_{n}\left(u\right)}$, it is straightforward to verify that

$$\begin{array}{c} {\hat{\Lambda }}_{n}\left(t\right)-\Lambda \left(t\right)=\int_{0}^{t}\frac{dH_{n}\left(u\right)}{R(u)}-\int_{0}^{t}\frac{R_{n}\left(u\right)}{R^{2}\left(u\right)}dH\left(u\right)+L_{n}\left(t\right), \end{array}$$

where

$$\begin{array}{c} L_{n}\left(t\right)=\;\;\int_{0}^{t}\left(\;\frac{1}{R_{n}\left(u\right)}-\frac{1}{R(u)}\;\right)d\left[H_{n}\left(u\right)-H\left(u\right)\right]+\;\;\int_{0}^{t}\frac{{\left(R_{n}\left(u\right)-R\left(u\right)\right)}^{2}}{R_{n}\left(u\right)R^{2}\left(u\right)}dH\left(u\right). \end{array}$$

Define $L_{n}\left(t\right)=:L_{n1}\left(t\right)+L_{n2}\left(t\right)$, where

$$\begin{array}{cc} L_{n1}\left(t\right) & =\int_{0}^{t}\left(\frac{1}{R_{n}\left(u\right)}-\frac{1}{R(u)}\right)d\left[H_{n}\left(u\right)-H\left(u\right)\right],\\ L_{n2}\left(t\right) & =\int_{0}^{t}\frac{{\left(R_{n}\left(u\right)-R\left(u\right)\right)}^{2}}{R_{n}\left(u\right)R^{2}\left(u\right)}dH\left(u\right). \end{array}$$

By Lemma (A.2), we have $\sup_{0<t<b}L_{n1}\left(t\right)=O\left(n^{-3/4}\log n\right),\;a.s$. Furthermore, by (A2), it follows that

$$\begin{array}{cc} \underset{0<t<b}{\sup}L_{n2}\left(t\right) & \leq \underset{0<t<b}{\sup}{\left(R_{n}\left(t\right)-R\left(t\right)\right)}^{2}\int_{0}^{b}\frac{dH(u)}{R_{n}\left(u\right)R^{2}\left(u\right)}\\ & =O\left(n^{-1}\log\log n\right)\;a.s. \end{array}$$

This completes the proof of the lemma.□

Proof of Proposition 2.1 Using the relation S(t)=exp{−Λ(t)}, and the approximation from Lemma A.1, with ${\bar{S}}_{n}\left(t\right)=\exp\left\{-{\hat{\Lambda }}_{n}\left(t\right)\right\}+O\left(n^{-1}\right)$, a.s., we have

$$\begin{array}{cc} {\hat{S}}^{W}\left(t\right)-S\left(t\right)= & \;{\bar{S}}_{n}\left(t\right)-S\left(t\right)+O_{p}\left(n^{-1}\right)\\ = & \;\exp\left\{-{\hat{\Lambda }}_{n}\left(t\right)\right\}-\exp\left\{-\Lambda \left(t\right)\right\}+O_{p}\left(n^{-1}\right), \end{array}$$

where ${\bar{S}}_{n}\left(t\right)=\exp\left\{-{\hat{\Lambda }}_{n}\left(t\right)\right\}$. By applying Taylor expansion and using Lemma A.3, we obtain

$$\begin{array}{cc} {\bar{S}}_{n}\left(t\right)-S\left(t\right)= & -\exp\left\{-\Lambda \left(t\right)\right\}\left({\hat{\Lambda }}_{n}\left(t\right)-\Lambda \left(t\right)\right)\\ & -\frac{\exp\{-\Lambda_{n}^{*}\left(t\right)\}}{2}{\left({\hat{\Lambda }}_{n}\left(t\right)-\Lambda \left(t\right)\right)}^{2}\\ = & -n^{-1}\sum_{i=1}^{n}S\left(t\right)\phi_{i}\left(t\right)+O\left(n^{-3/2}{\left(\log n\right)}^{2}\right)\;a.s., \end{array}$$

where $\Lambda_{n}^{*}\left(t\right)$ lies between $\min\{{\hat{\Lambda }}_{n}\left(t\right),\Lambda \left(t\right)\}$ and $\max\{{\hat{\Lambda }}_{n}\left(t\right),\Lambda \left(t\right)\}$. Thus, the proof of the theorem is complete.□

Proof of Lemma 4.3 Based on the representation in Proposition 2.1, the proposed pseudo‐observations ${\hat{S}}_{i}^{W}\left(t\right)$ can be simplified as

$$\begin{array}{cc} {\hat{S}}_{i}^{W}\left(t\right)= & \;n{\hat{S}}^{W}\left(t\right)-\left(n-1\right){\hat{S}}_{\left(-i\right)}^{W}\left(t\right)\\ = & \;nS\left(t\right)-\left(n-1\right)S\left(t\right)-\sum_{i=1}^{n}S\left(t\right)\phi_{i}\left(t\right)-\sum_{j\neq i}S\left(t\right)\phi_{j}\left(t\right)+o_{p}\left(1\right)\\ = & \;S\left(t\right)-S\left(t\right)\phi_{i}\left(t\right)+o_{p}\left(1\right), \end{array}$$

where $\phi_{i}\left(\cdot \right)$ is defined in (7). To evaluate $E({\hat{S}}_{i}^{W}\left(t\right)|X=x)$, we compute $E(\phi_{i}\left(t\right)|X=x)$. Define the martingale process $M_{i}\left(t\right)=N_{i}\left(t\right)-\frac{1}{2}\int_{0}^{t}R_{i}\left(u\right)d\Lambda \left(u\right)$, so that

(A4)
$$\begin{array}{c} \phi_{i}\left(t\right)=\int_{0}^{t}\frac{dM_{i}\left(u\right)}{R(u)}. \end{array}$$

Let the covariate‐specific version of $dM_{i}\left(t\right)$ be given by $dM_{i}\left(t|\tilde{X}=x\right)=dN_{i}\left(t\right)-\frac{1}{2}R_{i}\left(t\right)\lambda \left(t|\tilde{X}=x\right)dt$. Then,

(A5)
$$\begin{array}{c} dM_{i}\left(t\right)=dM_{i}\left(t|x\right)+\frac{1}{2}R_{i}\left(t\right)\left(\lambda \left(t|x\right)-\lambda \left(t\right)\right)dt. \end{array}$$

Under conditions (a) and (b), by simple calculation, we obtain

(A6)
$$\begin{array}{c} E\left(R_{i}\left(t\right)|X=x\right)=2\mu^{-1}S\left(t|x\right)w\left(t\right), \end{array}$$

Denote $R\left(t|x\right)=\frac{1}{2}E\left(R_{i}\left(t\right)|X=x\right)$. Then,

(A7)
$$\begin{array}{c} \frac{d}{dt}\left(\frac{R(t|x)}{R(t)}\right)=\frac{d}{dt}\left(\frac{S(t|x)}{S(t)}\right)=-\frac{R(t|x)}{R(t)}\left(\lambda \left(t|x\right)-\lambda \left(t\right)\right). \end{array}$$

Applying Equations (A4)–(A7), we derive

$$\begin{array}{cc} E[\phi_{i}\left(t\right)|X=x]= & \;\int_{0}^{t}\frac{1}{R(u)}E\left[dM_{i}\left(u|x\right)|X=x\right]\\ & +\frac{1}{2}\int_{0}^{t}\frac{1}{R(u)}E\left[R_{i}\left(u\right)|X=x\right]\left(\lambda \left(u|x\right)-\lambda \left(u\right)\right)du\\ = & \;\int_{0}^{t}\frac{\mu^{-1}S\left(u|x\right)w\left(u\right)}{R(u)}\left(\lambda \left(u|x\right)-\lambda \left(u\right)\right)du\\ = & \;\int_{0}^{t}\frac{R(t|x)}{R(t)}\left(\lambda \left(t|x\right)-\lambda \left(t\right)\right)dt\\ = & \;-\int_{0}^{t}\frac{d}{du}\left(\frac{S(u|x)}{S(u)}\right)du\\ = & \;1-\left(\frac{S(t|x)}{S(t)}\right). \end{array}$$

Therefore,

$$\begin{array}{c} E\left({\hat{S}}_{i}^{W}\left(t\right)|X=x\right)=S\left(t|x\right)+o_{p}\left(1\right), \end{array}$$

which completes the proof.□

Proof of Theorem 4.4 Under the notation in (14), we can rewrite

$$\begin{array}{c} \log\left(-\log S\left(t|x_{i}\right)\right)=\log\Lambda_{0}\left(t\right)+{x_{i}}^{⊺}\beta ={\overset{ˇ}{x}}_{i}^{⊤}\theta , \end{array}$$

or equivalently $S\left(t|x_{i}\right)=g^{-1}\left({\overset{ˇ}{x}}_{i}^{⊤}\theta \right)$, with the link function g(x)=log(−log(x)). From Lemma 4.3, we have

$$\begin{array}{c} E\left({\hat{S}}_{i}^{W}\left(t\right)|X_{i}=x_{i}\right)=S\left(t|x_{i}\right)+o_{p}\left(1\right)=g^{-1}\left({\overset{ˇ}{x}}^{⊤}\theta \right)+o_{p}\left(1\right). \end{array}$$

Thus, the estimating equation of the traditional generalized linear model for the longitudinal data ${\hat{S}}_{i}\left(t\right)$ can be written as

$$\begin{array}{c} U\left(\theta \right)=\sum_{i=1}^{n}{\left(\frac{\partial }{\partial \theta }E\left({\hat{S}}_{i}^{W}\left(t\right)|X_{i}\right)\right)}^{⊤}V_{i}^{-1}\left({\hat{S}}_{i}^{W}\left(t\right)-E\left({\hat{S}}_{i}^{W}\left(t\right)|X_{i}\right)\right)=0. \end{array}$$

By Theorem 1 of Liang and Zeger (1986), Theorem 4.4 is proved.□
