# Supplementary figures and images for: Pseudo‐Observation Approach for Length‐Biased Cox Proportional Hazards Model
Source: Biom J. 2025 Oct 30;67(6):e70094. doi: 10.1002/bimj.70094 (PMC12576048; doi:10.1002/bimj.70094)

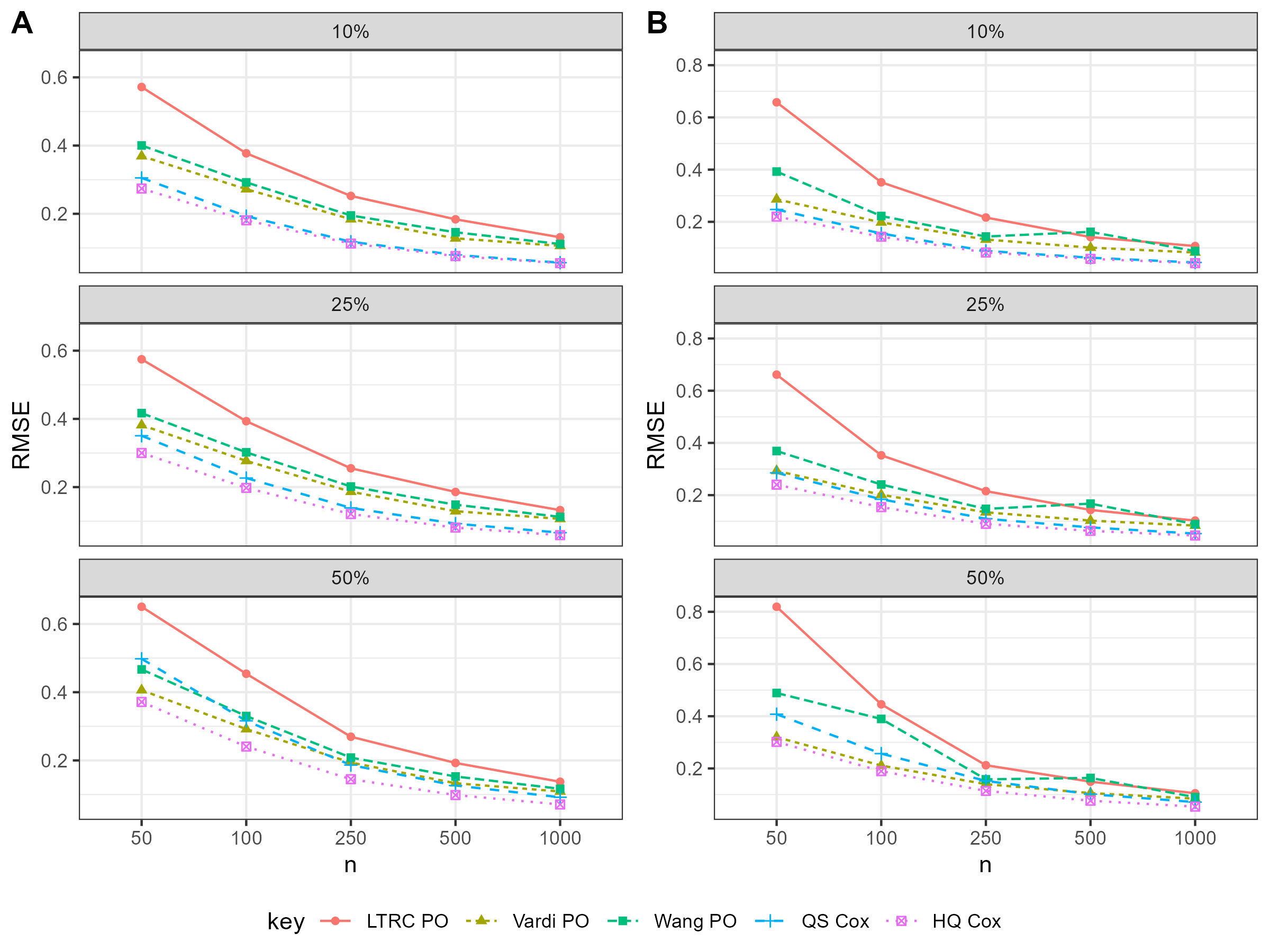

Supplement: Supplementary file 1 — Supporting Information [file BIMJ-67-e70094-s001.zip › code review round 2/results/Fig1.png]

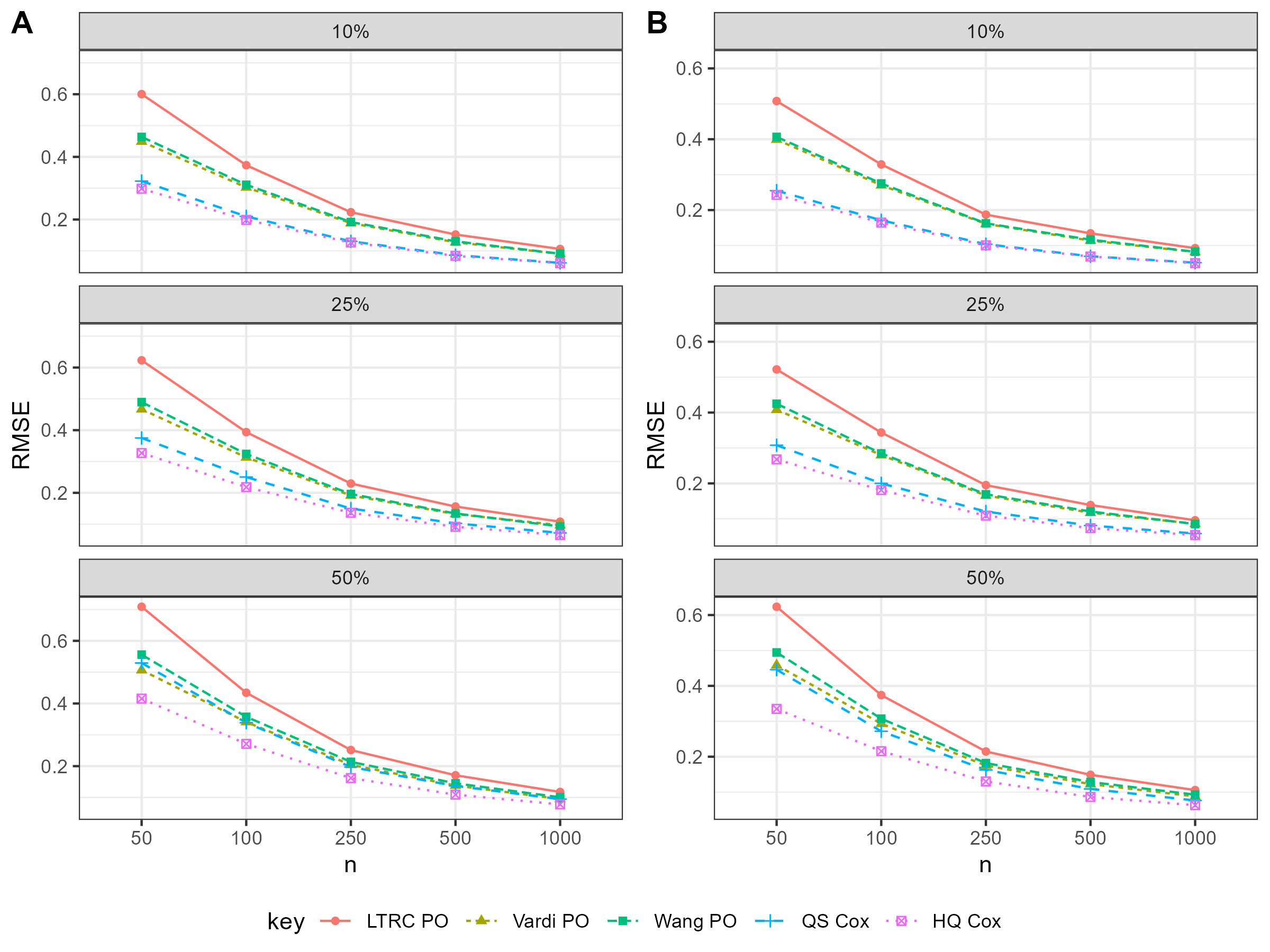

Supplement: Supplementary file 1 — Supporting Information [file BIMJ-67-e70094-s001.zip › code review round 2/results/Fig2.png]

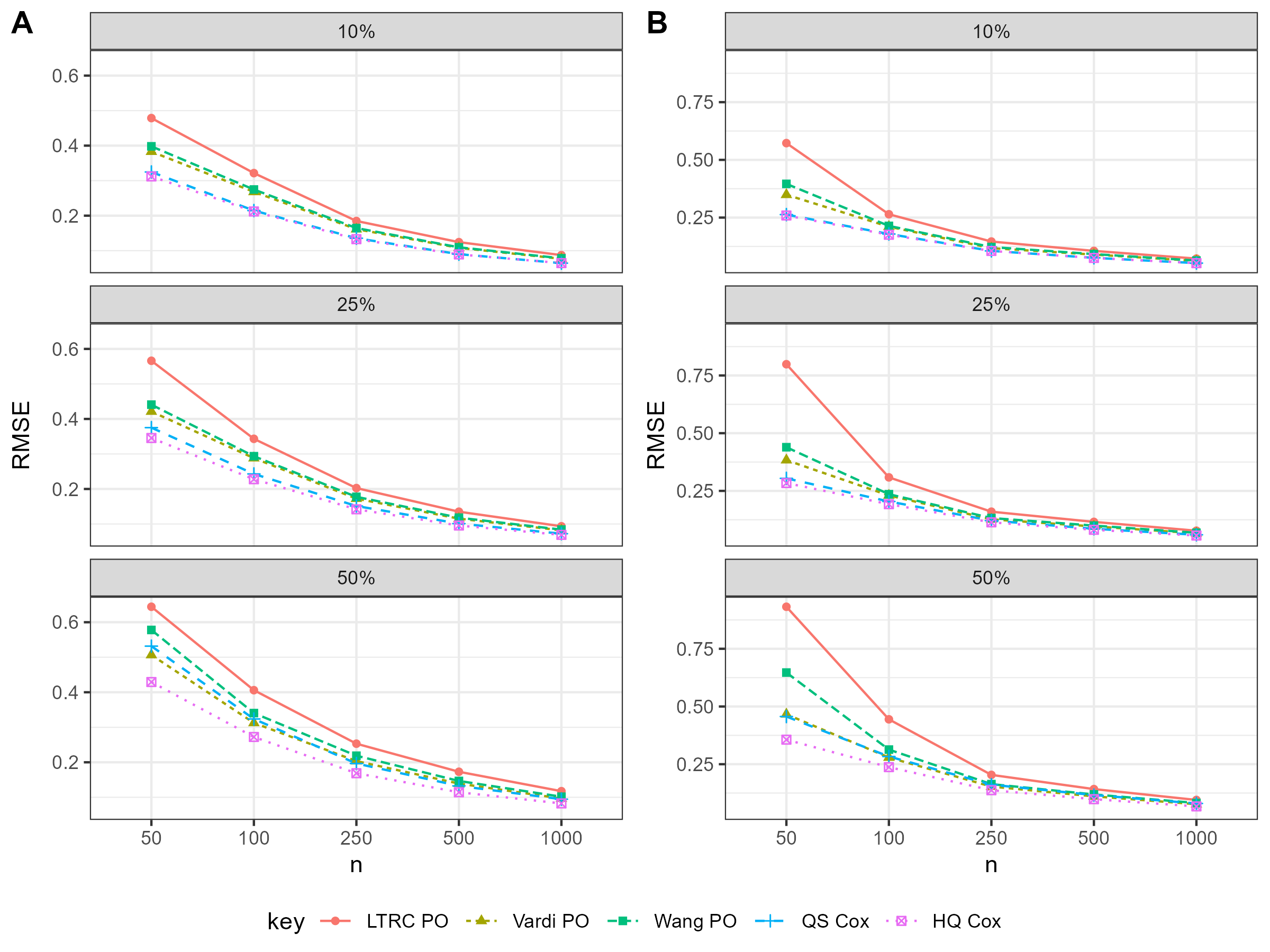

Supplement: Supplementary file 1 — Supporting Information [file BIMJ-67-e70094-s001.zip › code review round 2/results/Fig3.png]

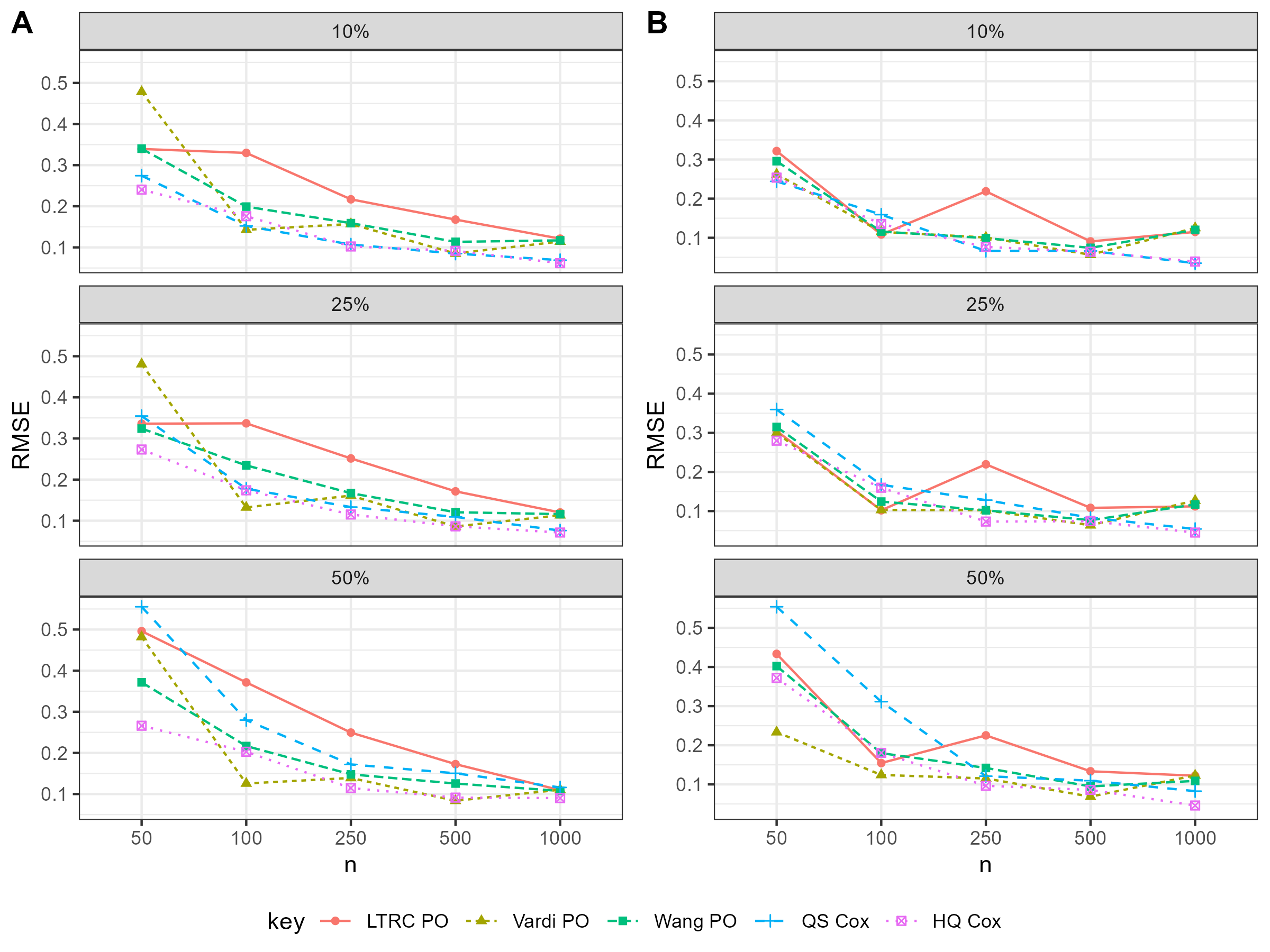

Supplement: Supplementary file 1 — Supporting Information [file BIMJ-67-e70094-s001.zip › code review round 2/simulation/intermediate_simulation/Fig1.png]
